# The Path to Group A *Streptococcus* Vaccines: World Health Organization Research and Development Technology Roadmap and Preferred Product Characteristics

**DOI:** 10.1093/cid/ciy1143

**Published:** 2019-01-08

**Authors:** Johan Vekemans, Fernando Gouvea-Reis, Jerome H Kim, Jean-Louis Excler, Pierre R Smeesters, Katherine L O’Brien, Chris A Van Beneden, Andrew C Steer, Jonathan R Carapetis, David C Kaslow

**Affiliations:** 1 Initiative for Vaccine Research, World Health Organization, Geneva, Switzerland; 2 International Vaccine Institute, Seoul, Republic of Korea; 3 Molecular Bacteriology Laboratory, Université Libre de Bruxelles; 4 Department of Pediatrics, Academic Children Hospital Queen Fabiola, Brussels, Belgium; 5 Tropical Diseases Research Group, Murdoch Children’s Research Institute; 6 Centre for International Child Health, University of Melbourne, Australia; 7 Johns Hopkins Bloomberg School of Public Health, Baltimore, Maryland; 8 Respiratory Diseases Branch, National Center for Immunization and Respiratory Diseases, Centers for Disease Control and Prevention, Atlanta, Georgia; 9 Department of Paediatrics, University of Melbourne; 10 Royal Children’s Hospital, Melbourne; 11 Telethon Kids Institute, University of Western Australia and Perth Children’s Hospital, Australia; 12 PATH, Seattle, Washington

**Keywords:** group A *Streptococcus*, vaccine, pharyngitis, rheumatic heart disease

## Abstract

Group A *Streptococcus* (GAS) infections result in a considerable underappreciated burden of acute and chronic disease globally. A 2018 World Health Assembly resolution calls for better control and prevention. Providing guidance on global health research needs is an important World Health Organization (WHO) activity, influencing prioritization of investments. Here, the role, status, and directions in GAS vaccines research are discussed. WHO preferred product characteristics and a research and development technology roadmap, briefly presented, offer an actionable framework for vaccine development to regulatory and policy decision making, availability, and use. GAS vaccines should be considered for global prevention of the range of clinical manifestations and associated antibiotic use. Impediments related to antigen diversity, safety concerns, and the difficulty to establish vaccine efficacy against rheumatic heart disease are discussed. Demonstration of vaccine efficacy against pharyngitis and skin infections constitutes a key near-term strategic goal. Investments and collaborative partnerships to diversify and advance vaccine candidates are needed.

## Teaser

Group A *Streptococcus* causes a wide spectrum of acute and chronic human disease and considerable antibiotic use. Through a consensus-building consultation process, the World Health Organization developed a vision for vaccine development, highlighting strategic targets, product preferences, and priority research/development activities.

Group A *Streptococcus* (GAS, *Streptococcus pyogenes*) is responsible for a wide range of acute and chronic clinical manifestations in humans. GAS infections and adverse consequences are estimated to cause about 0.5 million annual deaths, in all age ranges, mostly in young adults [1]. Yet, GAS has received little attention in global health programs, and existing tools for prevention are insufficient. In a 2018 resolution on rheumatic heart disease (RHD), a potential complication of GAS infections, the World Health Assembly highlighted the interest in GAS vaccines to complement control strategies [2].

The World Health Organization (WHO) provides guidance on research and development (R&D) priorities, to ensure that global health needs are addressed. The expression of priority objectives and activities can influence public and philanthropic investments, particularly when market incentives are insufficient to adequately drive private sector engagement [3].

Lack of relevant animal models, high genetic diversity of antigen targets, safety concerns, and lack of consensus on clinical endpoints for establishment of proof of concept have created major impediments to progress in GAS vaccine development to date [4]. Uncertain and/or insufficient market incentives are a remaining obstacle to private sector engagement, resulting in reliance on public and philanthropic investments to feed and advance the GAS vaccine pipeline. Following a WHO-sponsored consensus-building consultation process involving experts from academia, industry, funding bodies, regulatory agencies, and other government and public health organizations, strategic objectives and approaches to address existing impediments have been considered. In anticipation of requirements for regulatory and policy recommendations and to help define the value proposition for vaccines in development, WHO preferred product characteristics (PPC, an early development stage precursor to class- or product-specific target product profiles) [5] have been proposed and a research and development technology roadmap presented [5], briefly given here (Tables 1 and 2).

**Table 1.** Priority Activities as Expressed in the Vaccine Development Technology Roadmap for Group A *Streptococcus* Vaccines

Key Strategic Areas	Proposed Priority Activities
Research	Improve global estimates of disease burden and better characterize the epidemiology of GAS infections
	Further describe the spectrum of natural disease history
	Drive improved understanding of GAS-related secondary immune-mediated diseases
	Define the consequences of GAS-associated antibiotic use, and estimate the impact of vaccine use on antibiotic use and antimicrobial resistance–related morbidity and mortality
Vaccine development	Pursue antigen discovery efforts, increasing the number of pipeline vaccine candidates
	Develop consensus guidance about the appropriate use of safety monitoring tools in candidate vaccine trials
	Characterize immunological surrogates/correlates of protection
	Define appropriate pivotal clinical trial design adapted to near-term and long-term strategic goals
Key capacities	Define appropriate use of available and future animal models for GAS vaccine safety and efficacy evaluation according to their relevance for human responses
	Develop clinically relevant human GAS experimental infection model(s) to support early vaccine proof-of-concept evaluation
	Establish GAS expert research centers in LMICs with Good Clinical Practices trial research capacity and appropriate regulatory and ethical oversight; establish baseline rates of efficacy and safety outcomes
	Access low-cost vaccine manufacturing under current Good Manufacturing Practices for late-stage development and commercial production
	Develop standardized immunoassay platforms that meet quality requirements
Policy, commercialization, and delivery	Establish cost-effectiveness and develop research and implementation financial investment scenario(s) to support appropriate funding and policy decision making at the global and national levels, considering the full scope of costs and benefits
	Ensure availability, affordability, and acceptability of a functional, cost-effective delivery platform for immunization
	Develop effectiveness and safety vigilance platforms for postimplementation surveillance

Abbreviations: GAS, group A *Streptococcus*; LMICs, low- and middle-income countries.

**Table 2.** Preferred Product Characteristics for Group A *Streptococcus* Vaccines

Parameter	Preferred Characteristics
Indication	Prevention of GAS-related pharyngitis, superficial skin infections, cellulitis, toxin-mediated disease, invasive infections and associated antibiotic use, secondary rheumatic fever, rheumatic heart disease, and poststreptococcal glomerulonephritis
	*Notes:* Prevention of pharyngitis and skin infections would constitute relevant and feasible early vaccine development targets. See Efficacy section for further considerations on efficacy evaluation.
Target population for primary immunization	Primary schedule: infants and/or young children.
	*Notes:* Further evidence is needed to define the optimal vaccination age according to epidemiological setting, and whether GAS vaccination would be most appropriately introduced in early infancy, or require later, early childhood doses, and late booster doses. Research should determine the role of primary immunization in the following special circumstances: • Secondary prevention in subjects at increased risk of RHD • Immunization of adults at increased risk of cellulitis or severe invasive disease such as the elderly and individuals with diabetes, obesity, or other immunosuppressive conditions • Women, including pregnant women, for prevention of puerperal and neonatal sepsis • Immunization campaigns for interruption of outbreaks of GAS-related disease
Schedule of primary immunization and boosting	No more than 3 doses required for primary immunization.
	*Notes:* Research should determine the required number of doses and schedule for primary immunization and the requirements for booster doses. Boosting around school age, young adulthood and/or pregnancy, and old age could be proposed. Considering the age distribution of the disease burden, several booster doses may be required and acceptable.
Efficacy targets	Preferences for target efficacy differ according to the severity of the target disease syndrome: • 80% protection against nonsevere, noninvasive, confirmed GAS disease • 70% protection against confirmed GAS cellulitis and other invasive infections • 50% protection against long-term immune-mediated sequelae
	*Notes:* Lower limits of acceptable vaccine efficacy are not defined here. Long-term protection is required given the age distribution of the disease risk. The preferred minimum follow-up time for efficacy evaluation is 2 years. Appropriate efficacy endpoint case definitions and ascertainment methodologies for vaccine trials should be defined. The preferred efficacy thresholds for more severe outcomes are lower than those for less severe outcomes because of the public and individual value assessment. A strategy including predefined stage-gate criteria should be developed with the aim to minimize risk and accelerate vaccine development and to promote responsible research investment: • The availability of a clinically relevant human experimental infection model may be valuable. • Early proof of concept focusing on more frequent, less severe endpoints (with pharyngitis and skin infection as a priority) should establish the potential protective profile. • Vaccine efficacy against cellulitis and other invasive infections will require larger sample size. • The impact on longer-term, less frequent, severe complications may need to be evaluated in pilot implementation or postlicensure studies. The vaccine impact on carriage and transmission should be characterized.
Strain and serotype coverage	Efficacy targets are set irrespective of strain/serotype considerations. The vaccine composition should ensure that a vast majority (preference for at least 90%) of the current disease-causing isolates from the region targeted for use are prevented.
	*Notes:* The role of variation over time and potential for bacterial population replacement should be characterized. Further research is needed to determine role of immunoassays to infer strain/serotype specificity of protection.
Safety	Safety and reactogenicity profile at least as favorable as current WHO-recommended routine vaccines.
	*Notes:* As a minimum, a standard safety monitoring plan should be implemented as part of clinical development efforts. The appropriate use of additional safety monitoring tools including human antigen immune reactivity testing and echocardiography should be predefined, considering the risk of unspecific, coincidental findings, especially if multiple comparisons are planned. The intensity of safety investigations should be tailored to the amount of accrued evidence about the safety profile. Safety endpoints of interest should be protocol defined and supported by sample size analyses.
Adjuvant requirement	Evidence should be generated to justify adjuvant inclusion in the formulation.
	*Notes:* Adjuvants with established, favorable safety profiles are preferred over new adjuvants.
Immunogenicity	Established correlate/surrogate of protection based on a validated assay measuring immune effector levels/functionality.
	*Notes:* The longevity of the immune response should be characterized, and the relationship to duration of protection should be investigated. Collaborative efforts toward the generation of relevant nonclinical assays, using open source reference reagents (including immune sera) with international standards of quality may greatly contribute to comparability assessments, generation of a regulatory acceptable correlate of protection, ultimately supporting immune bridging steps and clinical development plan simplification, and accelerating the pathway to licensure. The role of reference laboratories is acknowledged.
Noninterference	Demonstration of favorable safety and immunologic noninterference upon coadministration with recommended other vaccines if used in the same target population.
Route of administration	Injectable (IM or SC) using standard volumes for injection as specified in programmatic suitability for PQ or needle-free delivery.
	*Notes:* The role of pain-free mucosal delivery via the pharynx or nasopharynx, and dermal delivery, should be considered. Preference for IM or SC over ID.
Registration, prequalification, and programmatic suitability	The vaccine should be prequalified according to the process outlined in Procedures for assessing the acceptability, in principle, of vaccines for purchase by United Nations agencies. WHO-defined criteria for programmatic suitability of vaccines should be met.
Value proposition	Dosage, regimen, and cost of goods amenable to affordable supply. The vaccine should be cost-effective and price should not be a barrier to access including in LMICs.
	*Notes:* Reduction of antibiotic use in routine practice would be of high added value. The vaccine impact on health systems, economic impact, and other aspects of implementation science should be evaluated in large trials, pre- or postapproval, as practicable.

Abbreviations: GAS, group A *Streptococcus*; ID, intradermal; IM, intramuscular; LMICs, low- and middle-income countries; PQ, prequalification; RHD, rheumatic heart disease; SC, subcutaneous; WHO, World Health Organization.

## BURDEN OF DISEASE AND CLINICAL DIVERSITY

GAS is a leading cause of infectious disease burden worldwide. The spectrum of GAS disease extends from superficial infections (eg, pharyngitis, impetigo), to invasive disease (eg, abscesses, cellulitis, sepsis), toxin-mediated disease (eg, scarlet fever, toxic shock syndrome, necrotizing fasciitis) and autoimmune sequelae (eg, acute rheumatic fever [ARF], poststreptococcal glomerulonephritis, and RHD). The most frequent manifestations are pharyngitis, with >616 million incident cases per year, and skin infections, with an estimated 162 million prevalent cases of impetigo [1, 6]. At least 18 million new cases of severe GAS diseases (RHD, ARF, glomerulonephritis, and invasive infections) are estimated to occur annually [1]. RHD alone is responsible for a very large burden of chronic disability and deaths, mostly in adolescents and young adults, particularly pregnant women. The global prevalence of RHD cases was estimated to be 33 million in 2015 [7].

Globally, GAS disease and its complications have been reported to cause 500 000 annual deaths, of which 319 000 are due to RHD [1]. Populations from low- and middle-income countries (LMICs) are at greatest risk. Global mortality due to RHD has somewhat declined since 1990, but no significant decline has been observed in the regions that carry the highest disease burden [7, 8]. Gaps in data availability hinder the accuracy of global burden estimates. The scarcity of disease registries in most LMICs, reliance on passive surveillance systems, and underreporting of cases continue to be challenging [7, 8].

Timely and targeted antibiotic treatment of GAS infections constitutes the backbone of prevention of complications [9]. The delivery of preventive interventions has been difficult in settings with fragile health systems and limited access to care, and insufficient to show major impacts [7]. While high-income countries (HICs) have managed to massively reduce the RHD burden, other manifestations of GAS infection—sepsis, cellulitis, necrotizing fasciitis, and toxic shock syndrome—remain prevalent. The United Kingdom is witnessing a surge in scarlet fever outbreaks as well as increasing incidence of invasive GAS infections [10], also reported in the United States and Canada [11, 12]. Among invasive disease cases, there is a specific maternal and early life disease burden in both LMICs and HICs [13, 14]. Contributors to adverse outcomes include delay in recognizing severity and initiating treatment, and restrictions in availability of first-line injectable antibiotics [13, 14].

GAS is also an important driver of antibiotic use [15]. To manage acute infections and avert complications, sore throat and skin infections are often treated with antibiotics, in a way frequently inconsistent with guidelines. Most pharyngitis cases are due to viruses [16], but data from surveys of ambulatory practice in the United States show that 60% of consultations for sore throat lead to antibiotic prescription for both children and adults [15–17]. Broad-spectrum antibiotics are often unnecessarily used [17]. Over-the-counter overuse of antibiotics is a significant problem in many LMICs [18]. While GAS remains universally susceptible to penicillin, antibiotic exposure of the commensal flora contributes to long-term dysbiosis and emergence of antibiotic resistance, a growing public health crisis [18].

## IMMUNITY TO GAS AND MOLECULAR EPIDEMIOLOGY

A vaccine against GAS could reduce the related burden on individuals, communities, health systems, and societies as a whole and, through a reduction of antibiotic use, help contain antimicrobial resistance and reduce dysbiosis.

A better understanding of the determinants of immunity in conditions of natural exposure may help guide development strategies. The observation that noninvasive infections are much more common in children than in adults suggests that natural exposure may generate partial immunity. The current assumption is that repeated infections with different serotypes lead to partial, antibody-mediated cross-strain protection [19]. Adults have higher levels of circulating anti-GAS antibodies compared with children [20]. The incidence of invasive and other severe GAS infections is also higher in young children than in young and middle-aged adults. The increased disease rates in the elderly may be related to immunosenescence and prevalence of comorbidities [21]. Antibodies may also bind and neutralize streptococcal toxins, such as streptococcal pyrogenic exotoxins A, B, and C and erythrogenic exotoxin B [22].

There is presently limited knowledge about the immune determinants of GAS carriage. It was shown for other bacterial vaccines that reduction in carriage and transmission was an important driver of impact [23]. A better understanding of the contribution of mucosal and systemic immunity in preventing surface colonization and invasive infections could provide critical insights into how to optimally deploy GAS vaccines to maximize the population-based benefits and cost-effectiveness, particularly in LMIC settings. Immunoepidemiologic studies may also provide insights into mediators of acquired immunity following natural exposure.

Another major determinant of impact of GAS vaccines will be the breadth of responses against immunodominant target antigens displaying a high degree of genetic diversity. The *emm* gene–encoded M protein on the bacterial surface is a major virulence and immunologic determinant [24]. M typing has been the priority approach to GAS global molecular diversity characterization. Approximately 50 different serotypes were first identified [25, 26]. More recently, molecular biology techniques supported *emm* type classification, further grouped into *emm* clusters according to entire M protein sequences and related biological properties [27].

The global distribution of GAS *emm* types is extremely diverse: *emm*1 and *emm*12 are the 2 most common types in Asia, Latin America, and the wealthiest countries, which have the lowest strain diversity. These predominant strains in HICs are less prevalent in Africa and the Pacific regions, where there is greater strain diversity [24]. Diversity likely depends on several factors, including social determinants, as illustrated by a study in the city of Salvador in Brazil, showing that strain diversity in a slum was greater than in neighboring high-income suburbs [28].

The extensive *emm* type diversity poses challenges for the development of M protein–specific vaccines. Sequences selected according to prevalent strains in HICs might confer poor coverage in high-burden regions [24]. Although cross-opsonic antibodies against nonvaccine serotypes have been demonstrated in vitro following vaccination with a 30-valent M protein–based vaccine, clinical significance remains to be determined [29]. Further characterization of GAS isolates and epidemiologic distribution is needed to help guide rational vaccine development [24]. Conserved antigen discovery efforts are needed to improve the current vaccine pipeline.

## VACCINE PIPELINE

Only 2 candidate vaccines are actively under evaluation in human trials. A phase 1 clinical trial of the MJ8VAX vaccine candidate developed by the Queensland Institute of Medical Research, Australia, was recently reported. The vaccine antigen is a 29-amino-acid–long peptide (J8) from the conserved carboxyl terminus region of the M protein [30], conjugated with diphtheria toxoid and adsorbed onto aluminium hydroxide. More investigations are planned to further optimize immunogenicity.

The 30-valent StreptAnova, developed at the University of Tennessee and at Dalhousie University, Canada, is an M protein–based vaccine with 4 recombinant subunits, each containing 7 or 8 N-terminal fragments of 30 different *emm* types linked in tandem [29]. The N-terminal fragment of the Spa18 antigen is also included in the construct. The peptides were selected from acute and invasive isolates most prevalent in North America and Europe. A phase 1 clinical trial of the vaccine adjuvanted with alum was recently completed. This program builds on favorable safety and immunogenicity evaluation of previous related constructs including a lower number of *emm* type sequences [29].

In preclinical development, the StreptIncor vaccine candidate construct developed by the University of São Paulo, Brazil, is based on the conserved region of the M5 protein, which comprises a 55-amino acid polypeptide containing conserved B- and T-cell epitopes. A phase 1/2a clinical trial of the vaccine candidate antigen formulated with alum is expected to start in 2018–2019 [31]. Investments in GAS vaccine R&D by major vaccine manufacturers have been limited. One candidate based on the conserved antigens streptolysin O, SpyAD, SpyCEP, and group A carbohydrate conjugated with a carrier protein is being developed by GlaxoSmithKline [32]. The antigens selected are highly conserved and prevalent, either surface-exposed or secreted, expressed during human infection, soluble, and immunogenic in animals.

Altogether, the scarcity of products in development as presented above underscore the need to expand and diversify the vaccine pipeline.

## SAFETY CONSIDERATIONS

Safety concerns have constituted an important impediment to past vaccine development efforts. In 1969, the occurrence of ARF following streptococcal vaccination in 3 of 21 volunteers vaccinated with a partially purified M3 protein was reported [33]. This raised concerns about the safety of GAS vaccines and a theoretical risk of autoimmunity. In 1979, the US Food and Drug Administration (FDA) prohibited the use of GAS organisms and their derivatives in any bacterial vaccine [34]. However, the validity of such concerns raised by this single study was subsequently questioned. All 3 children had documented GAS infection before the onset of ARF, and all were siblings of ARF patients. They were exposed to very high and repeated dosing of a crude M protein vaccine formulation. These factors may have influenced their risk of developing ARF. The FDA resolution was revoked in 2006, when the agency recognized the previous understanding as “both obsolete and a perceived impediment to the development of a GAS vaccine” [34]. There had not been a GAS vaccine trial reported during a period of 25 years. Vaccine research resumed, with no similar adverse safety signal identified.

Nonetheless, the field would benefit from consensus building on safety risk management strategies appropriately adapted to vaccine development status. Studies have often used serum autoimmunity panel screening, tissue cross-reactive immunofluorescence antibody assays, and echocardiography to monitor for potential autoimmune events occurring postvaccination [30]. While due diligence is needed, there is a strong perception that autoantibody panels and echocardiographic monitoring are poor screening tools and of limited value, as adequate safety monitoring requires sufficient endpoint sensitivity and specificity, especially when the number of trial participants is limited as in early vaccine development. In clinical practice, these tests are seldom used in isolation, as their contribution to diagnosis is strongly driven by pretest probability determined by the clinical context. Borderline results and nonspecific findings make interpretation difficult [35]. Screening panels and echocardiographic evaluation may best be reserved for screening out subjects at increased risk of abnormalities detected before entering into investigational vaccine studies.

## PRECLINICAL TOOLS, EARLY ESTABLISHMENT OF CLINICAL PROOF OF CONCEPT, AND LONG-TERM GOALS

GAS is strictly a human pathogen, and many of its virulence factors are only active against human cells and proteins. While animal models have been developed and used to study GAS pathogenesis and GAS vaccine candidates, the wide variety of clinical manifestations is a challenge for the development of a relevant and representative animal disease model [36]. The high level of strain diversity is another barrier, as no single strain can be considered representative of the bacterial population as a whole and only a few strains have been demonstrated to be virulent in different animal models [37]. Therefore, despite being useful for preclinical toxicity studies and as screening tools, the overall role of animal studies in the development of a GAS vaccine might be limited.

In the absence of relevant animal models, the availability of early strategic development milestones and proof-of-concept efficacy endpoints are essential to optimally manage investment risk. Controlled human infection models, when available, provide the ability to fail fast and early, a key asset in resource management, and constitute a powerful tool to dissect pathogenesis and immune protective mechanisms, and establish correlates of protection [4].

The difficulty to demonstrate vaccine efficacy against RHD, a major but distant outcome, has been an important impediment to vaccine development efforts. To address this, clinical development pathways involving early demonstration of vaccine efficacy against GAS pharyngitis and skin infections are being proposed, as illustrated by the near-term strategic goal expressed in the R&D technology roadmap (Table 3). Pharyngitis and skin infections have a high incidence in children, are globally distributed, and are responsible for widespread antibiotic use [15–17, 38]. Evidence shows that these infections are on the obligatory causal pathway to many of the severe outcomes of GAS infections, including invasive infections, and RHD [39]. Prevention of these noninvasive infections likely would also prevent disease progression and long-term consequences. This approach is compatible with a clinical development plan proposed by key stakeholders in GAS vaccine development [35]. A specific difficulty for the demonstration of vaccine efficacy against GAS skin infections relates to the frequent association with scabies, especially in tropical areas. Implications on the vaccine evaluations should be further considered [38].

**Table 3.** Strategic Goals

Near-term Strategic Goals	Long-term Strategic Goals
To demonstrate favorable safety and proof of efficacy of candidate vaccines against group A *Streptococcus* pharyngitis and skin infections in children.	To develop safe, globally effective, and affordable group A *Streptococcus* vaccines for prevention of acute infections (pharyngitis, skin infections, cellulitis, invasive disease) and associated antibiotic use and secondary immune-mediated sequelae (kidney disease, rheumatic fever, and rheumatic heart disease) and associated mortality.

Long-term goals relate to the evaluation of the full scope of the public health benefit provided by a vaccine. Depending on the disease entity or impact criteria considered, evidence could be generated from large phase 3 trials conducted conditionally upon successful achievement of short-term strategic goals. Alternatively, prevention of some disease entities may need to be investigated postlicensure, in pilot demonstration probe studies, as is increasingly becoming necessary for effectiveness and health economic vaccine evaluation.

## PREFERRED PRODUCT CHARACTERISTICS

The WHO PPC documents provide guidance as to WHO’s preferences for new vaccines in priority disease areas, promote the development of vaccines with optimal effectiveness and suitability for use in LMICs, and help define the value proposition of LMIC markets for vaccines in development, subsequently informing class- or product-specific target product profiles. WHO PPCs aim to support research in areas of public health need, setting realistic expectations about key characteristics likely to be supportive of positive decision making. The WHO PPC documents do not aim to express minimal acceptable criteria, but rather to express aspirational goals whereas target product profiles, which are a mainstay of industry, typically set out product-specific target performance criteria for a specific vaccine candidate. Any locally licensed product could be considered for WHO prequalification and policy decision making according to the defined process, even if expressed preferences are not met, as PPCs do not constitute formal guidance and do not preempt any regulatory reviews or policy decisions.

In brief, as introduced above, although proof of concept will likely rely on the demonstration of efficacy against pharyngitis and skin infections, long-term evaluation, possibly post–initial licensure, should provide evidence of protection against a larger spectrum of disease. Young children should be vaccinated before the peak incidence of GAS pharyngitis, but, in perspective of the future of immunization practices, a life-course strategy including boosters should be envisaged, as the global burden, especially when considering invasive infections, is widely distributed across age categories. Preferred efficacy targets reflect the perceived value of required investments, the value of such investments being higher when the targeted outcome is more severe.

An abbreviated version of the WHO PPCs for GAS vaccines is presented in Table 2, with high-level explanatory notes providing additional considerations about preferred attributes. For full details, readers are encouraged to consult the source reference [5].

## R&D TECHNOLOGY ROADMAP

Fifteen years after the first global burden estimates highlighting the major GAS-related worldwide disease burden, the field remains significantly underfunded, and limited resources have been directed to support vaccine development efforts. The lack of consensus on what constitutes the major scientific gaps and how to address them, and an unclear vision of the value of investments in GAS vaccine development, has hindered governments, funders, and manufacturers, to prioritize seed investments. The expression of a strategic vision for vaccine development as presented in an R&D technology roadmap aims to influence and guide decision making in this long-neglected area. Priority activity areas are proposed. Background research should further establish global epidemiological features, the natural disease history, and determinants of adverse immunologic outcomes. Key capacities, disease models, and immunologic and safety monitoring tools should be further developed to support vaccine clinical testing in all geographical areas. An abbreviated version of the roadmap is presented in Table 1. For full details, readers are encouraged to consult the source reference [5].

## THE POWER OF PARTNERSHIPS, ADVOCACY, AND VALUE PROPOSITION ANALYSIS

The establishment of a collaborative partnership network to lead the implementation of the WHO GAS Vaccine Development Technology Roadmap, in line with the vision expressed in the GAS Vaccine PPC document, could guide, foster, and accelerate progress in GAS vaccine development and introduction. One priority deliverable of such partnership would be a comprehensive analysis of the full public value of GAS vaccines, considering the clinical as well as the socioeconomic, regulatory, policy, delivery, and user perspectives. This full public value proposition would inform different stakeholders about how a potential GAS vaccine could meet their interests and needs, guiding better-informed investments, mobilizing capacities in a rational way, and increasing predictability on the pathway to this much-needed vaccine.

## Notes

***Acknowledgments.* **The authors are grateful to the Wellcome Trust, particularly Zoe Seager and Sushmita Sarkar, for support in the organization of the group A *Streptococcus* vaccine World Health Organization (WHO) consultation in London in May 2018, and to all the individuals who participated.

***Financial support.* **The Wellcome Trust has contributed in-kind to support the WHO consultation processes described here.

***Potential conflicts of interest.* **All authors: No reported conflicts. All authors have submitted the ICMJE Form for Disclosure of Potential Conflicts of Interest. Conflicts that the editors consider relevant to the content of the manuscript have been disclosed. The authors declare no conflicts of interest.
